# Ozone Impact on Emission of Biogenic Volatile Organic Compounds in Three Tropical Tree Species From the Atlantic Forest Remnants in Southeast Brazil

**DOI:** 10.3389/fpls.2022.879039

**Published:** 2022-06-24

**Authors:** Bárbara Baêsso Moura, Vanessa Palermo Bolsoni, Monica Dias de Paula, Gustavo Muniz Dias, Silvia Ribeiro de Souza

**Affiliations:** ^1^Institute of Research on Terrestrial Ecosystems, National Research Council of Italy, Sesto Fiorentino, Italy; ^2^Núcleo de Uso Sustentável de Recursos Naturais, Instituto de Pesquisas Ambientais de São Paulo, São Paulo, Brazil; ^3^Centro de Ciências Naturais e Humanas, Universidade Federal do ABC, Santo André, Brazil

**Keywords:** ecological chemical trait, tropospheric ozone, VOC, tropical plants, isoprenoid compounds

## Abstract

Plants emit a broad number of Biogenic Volatile Organic Compounds (BVOCs) that can impact urban ozone (O_3_) production. Conversely, the O_3_ is a phytotoxic pollutant that causes unknown alterations in BVOC emissions from native plants. In this sense, here, we characterized the constitutive and O_3_-induced BVOCs for two (2dO_3_) and four (4dO_3_) days of exposure (O_3_ dose 80 ppb) and evaluated the O_3_ response by histochemical techniques to detect programmed cell death (PCD) and hydrogen peroxide (H_2_O_2_) in three Brazilian native species. *Croton floribundus* Spreng, *Astronium graveolens* Jacq, and *Piptadenia gonoacantha* (Mart.) JF Macbr, from different groups of ecological succession (acquisitive and conservative), different carbon-saving defense strategies, and specific BVOC emissions. The three species emitted a very diverse BVOC composition: monoterpenes (MON), sesquiterpenes (SEQ), green leaf volatiles (GLV), and other compounds (OTC). *C. floribundus* is more acquisitive than *A. graveolens*. Their most representative BVOCs were methyl salicylate—MeSA (OTC), (Z) 3-hexenal, and (E)-2-hexenal (GLV), γ-elemene and (−)-β-bourbonene (SEQ) β-phellandrene and D-limonene (MON), while in *A. graveolens* were nonanal and decanal (OTC), and α-pinene (MON). *Piptadenia gonoachanta* is more conservative, and the BVOC blend was limited to MeSA (OTC), (E)-2-hexenal (GLV), and β-Phellandrene (MON). The O_3_ affected BVOCs and histochemical traits of the three species in different ways. *Croton floribundus* was the most O_3_ tolerant species and considered as an SEQ emitter. It efficiently reacted to O_3_ stress after 2dO_3,_ verified by a high alteration of BVOC emission, the emergence of the compounds such as α-Ionone and trans-ß-Ionone, and the absence of H_2_O_2_ detection. On the contrary, *A. graveolens*, a MON-emitter, was affected by 2dO_3_ and 4dO_3_, showing increasing emissions of α-pinene and β-myrcene, (MON), γ-muurolene and β-cadinene (SEQ) and H_2_O_2_ accumulation. *Piptadenia gonoachanta* was the most sensitive and did not respond to BVOCs emission, but PCD and H_2_O_2_ were highly evidenced. Our results indicate that the BVOC blend emission, combined with histochemical observations, is a powerful tool to confirm the species’ tolerance to O_3_. Furthermore, our findings suggest that BVOC emission is a trade-off associated with different resource strategies of species indicated by the changes in the quality and quantity of BVOC emission for each species.

## Introduction

The ozone (O_3_) is a phytotoxic air pollutant that extensively affects plant physiology (Ashmore, 2005; Juráň et al., 2021). Its impact on Biogenic Volatile Organic Compounds (BVOCs) emission has been reported in several native and crop species (Pinto et al., 2010; Acton et al., 2018). The O_3_ levels critical for vegetation are commonly exceeded in North America, East Asia, and Europe (CLRTAP, 2017). In South America, instead, O_3_ levels tend to be lower (Erickson et al., 2020). However, O_3_ levels have been confirmed to cause injury to the foliage of native tree species representative of the Atlantic Forest remnants. In addition, the accumulated seasonal exposure over 40 ppb hourly concentrations (AOT40) exceeds the UNECE critical threshold of 5 ppm h in several monitoring stations located near the Atlantic Forest remnants (Moura et al., 2014), reinforcing the need to understand how vegetation responds to changes in air quality, especially in those developing regions where O_3_ monitoring is insufficient.

The O_3_ acts inside the leaves; it enters through the stomata and quickly degrades, reacting in the apoplast to form reactive species of oxygen (ROS), which oxidize cellular constituents, such as lipids, proteins, and nucleic acids (Overmyer et al., 2009). The oxidative stress occurs only when the imbalance between ROS production and the detoxification process occurs (Baier et al., 2005); thus, the capacity to scavenge ROS can be directly related to the species’ sensibility. Among the ROS, hydrogen peroxide (H_2_O_2_) is a remarkably stable molecule with no electric charge and free diffusion between cell walls and membranes (Iriti and Faoro, 2008). Additionally, H_2_O_2_ may act as a signaling molecule able to either trigger antioxidative defenses or produce oxidative stress activating a programmed cell death (PCD) process (Pellinen et al., 1999). Histochemical tests have been successfully applied in studies of oxidative stress in plants (Alves et al., 2011; Moura et al., 2014) and have been recognized as essential tools to detect H_2_O_2_ accumulation before the PCD (Faoro and Iriti, 2005; Turc et al., 2021).

Plants emit many BVOCs, covering up to 1700 known compounds (Dudareva et al., 2006, 2013). BVOCs are produced in different plant tissues by diverse physiological processes. They are mainly leaf-constitutive, effective in tri-trophic interaction, or produced in the flowers and related to the pollination process as attractors to pollinators and seed dispersers (Kesselmeier and Staudt, 1999; Arimura et al., 2009; Holopainen and Gershenzon, 2010; Dudareva et al., 2013). Among the BVOCs produced by plants, the isoprenoids, in particular the isoprene, monoterpenes (MON), sesquiterpenes (SEQ), and green leaves volatiles (GLV), have been extensively investigated (Peñuelas and Staudt, 2010; Guenther, 2013).

The BVOC emission rates can vary depending on plant species and environmental conditions, such as temperature, solar radiation, humidity, seasonality, and atmospheric pollution (Wang et al., 2021). Also, it is strongly dependent on the stressor agent (Arimura et al., 2009; Holopainen and Gershenzon, 2010; Peron et al., 2021) such as wounding, herbivory, water deficit, and O_3_. The BVOCs play a crucial role in protecting plants against abiotic and biotic stress due to the chemical signaling of the plant defense system (Vickers et al., 2009). The most important BVOC subsets involved in plant defense are MON, SEQ, and GLV (Niinemets et al., 2004), which can increase by several orders of magnitude when plants are under stress (Peñuelas and Llusià, 1999; Loreto and Velikova, 2001). Thus, the stress sensitivity of BVOC emission can provide important information about plant responses to environmental stresses.

Based on the concentration, the duration of the exposure, and the responsiveness of the plant species, O_3_ can induce the emission of a different bouquet of BVOCs (Roshchina and Roshchina, 2013; Buchanan et al., 2015; Peron et al., 2021; Yuan et al., 2021). Therefore, atmospheric O_3_ rise can be an important abiotic stressor, which significantly influences BVOC’s global emissions and plays a crucial role in many aspects of environmental science. On the other hand, BVOCs substantially impact atmospheric chemistry, participating in O_3_ production and aerosol formation (Coggona et al., 2021). Indeed, they act as mediators in the tropospheric interactions in terrestrial ecosystems with multiple functions, such as defense against herbivores and pathogens, and play a signaling role in plant–plant communication (Meents and Mithöfer, 2020; Ninkovic et al., 2021).

Although the knowledge about the importance of BVOCs in relation to global climate change is well studied (Laothawornkitkul et al., 2009; Peñuelas and Staudt, 2010; Lun et al., 2020; Liu et al., 2021), the induction of BVOCs by O_3_ is still poorly known (Pinto et al., 2010; Li et al., 2017), especially for tropical species (Kesselmeier et al., 2013; Yáñez-Serrano et al., 2020). Studies that report the O_3_ effect on BVOCs emission, biochemical, and morphological plant responses of plant communities, and ecosystem degradation usually focus on species from the northern hemisphere (Hartikainen et al., 2009; Kulmala et al., 2013).

The data are scarce in the Southern Hemisphere, including the Atlantic Forest. However, our previous studies of *Croton floribundus* Spreng have demonstrated that high O_3_ induces the BVOCs emission and consequently increases the calcium crystal formation (Cardoso-Gustavson et al., 2014; Bison et al., 2018). Moreover, *C. floribundus* Spreng., *Piptadenia gonoacantha* (Mart.) J. F Macbr. and *Astronium graveolens* Jacq, representative species of Atlantic Forest remnants of São Paulo-southeast Brazil, are well investigated and have visual symptoms described in experimental and field conditions (Moura et al., 2014, 2018). Physiological (Cassimiro et al., 2016; Fernandes and Moura, 2021) and biochemical responses (Domingos et al., 2015) are also well described and point to the use of these species for future biomonitoring of O_3_ potential effects in tropical regions. These three plant species have shown different biochemical strategies for their defense responses. *C. floribundus* is the most O_3_-tolerant, following *A. graveolens* (Brito et al., 2021) and *P. gonoachanta* (Esposito et al., 2018), which is in accordance with their ecological succession, from acquisitive to conservative as acquisitive species present a higher carbon reserve than conservative species and thus high tolerances for abiotic stress (Fichtner et al., 2017). Thus, we hypothesized that the complexity of BVOCs emitted by these species is related to their ecological function. The acquisitive species are more isoprenoid-emitters with greater carbon accumulation, which can be a functional trait; however, the effect of O_3_ can change BVOC emission, and its ecological functionality leads to still unknown consequences in the ecosystem.

In the present study, we selected model plants widely distributed in the urban secondary forest of the São Paulo Metropolitan region, Brazil. These plants are not considered at risk of extinction but are strongly affected by O_3_. Therefore, this work aimed to investigate the BVOCs profile emission rate of these tropical plant species under high O_3_ concentrations and verify the emission changes when exposed to an O_3_ enriched environment, identifying specific BVOCs emitted as stress indicators of oxidative conditions. The assessment of O_3_ impact on BVOC emission from tropical native species is necessary to understand how BVOCs can feedback the tropospheric O_3_ and contribute to secondary aerosol formation and, consequently, their effects on climate change.

## Materials and Methods

### Plant Material

Seedlings of *C. floribundus* Spreng., *Piptadenia gonoacantha* (Mart.) Macbr. and *Astronium graveolens* Jacq. were acquired commercially (nursery Bioflora, São Paulo, Brazil). The individuals were standardized by height (approximately 50 cm) and transplanted to 5 L plastic pots containing Eucatex Plantmax substrate mixed with vermiculite (3:1). The plants were maintained for 1 month in a greenhouse with filtered air and capillary irrigation and received 100 ml of Hoagland nutrient solution (Epstein, 1975).

### O_3_ Exposure

Twenty-seven plants of each species were selected and acclimated to internal conditions of light (average radiation: 422 μmol cm^2^sˉ^1^, provided by metallic vapor—400 W and fluorescent - 30 W TL05 lights), temperature (27 ± 2°C), and humidity (67.3 ± 5.2%) for 2 days of the fumigation system at the Atmosphere-Plant Interaction Laboratory (LABIAP) of the Ecology Research Center. Plants were transferred from the greenhouse to the fumigation chambers, where they were kept for 2 days before the beginning of the fumigation experiment (acclimation period). The O_3_ exposure was performed during the summer of 2018. The chambers were programmed to have a light (L): dark (D) cycle of 10 h L:14 h D (Pedrosa et al., 2020). Plants were exposed to 80 ppb of O_3_ for 5 h day from 8 AM to 13 PM. The O_3_ level was chosen according to the daily average of O_3_ in São Paulo for two decades (Schuch et al., 2019). All details about the fumigation facilities are described in Souza and Pagliuso (2009).

Nine individuals of each species were exposed per treatment. Two treatments were carried out: Filtered air, denoted as control (CT), and Filtered air enriched with 80 ppb of O_3_ for 2 (2dO_3_) and 4 (4dO_3_) days of exposure. Three replicates of each exposure were made for the three studied species. Each replicate was done simultaneously with its control in the same week. The three replicates (*n* = 9 individuals per replicate) of each species were sampled at the end of the experiment.

### Histochemical Tests

The PCD was assessed with Evans blue staining, performed by boiling leaf samples (1 cm^2^) for 1 min in a mixture of phenol, lactic acid, glycerol, and distilled water containing 20 mg mL^−1^ Evans’s blue (1:1:1:1) according to Iriti and Faoro (2003). This mixture was prepared immediately before use. Tissues were then clarified overnight in 95% ethanol (Iriti and Faoro, 2003 modified). Dead cells were stained from dark to light blue, depending on the stage of cell membrane degradation (Faoro and Iriti, 2005), while intact cells did not stain.

For the detection of H_2_O_2_, samples (1 cm^2^) of fresh leaves were immersed in 3,30′-diaminobenzidine (DAB), adjusted to pH 5.6 with NaOH, and incubated in a growth chamber for 8 h in the dark. Samples were then cleared in 96% ethanol (Faoro et al., 2001; Faoro and Iriti, 2005). The H_2_O_2_ was visualized as a reddish-brown color. As a negative control, the DAB solution was supplemented with 10 mM ascorbic acid (Faoro et al., 2001). All samples were examined with an Olympus BX41 light microscope (Tokyo, Japan) equipped with a digital camera (Media Cybernetics PL-A624, Bethesda, MD, United States).

Considering the known homogeneity in the distribution of O_3_ injury in the leaves of the species studied (Moura et al., 2018) and in order to have a representative evaluation of all individuals, five samples were randomly selected from three leaves of each species per treatment (CT and 4dO_3_) were processed and analyzed.

### Biogenic Volatile Organic Compounds: Sampling and Analysis

For the BVOC sampling, branches of each individual were enclosed in bags homemade with Teflon film (50 μm thickness, Dupont, United States). Four Teflon Bags were flushed with O_3_-free ambient air. One was used as the reference “empty,” and the others enclosed a branch above ground. Ambient air was scrubbed of particulate matter using PFE filters (Teflon Filter, 47 mn, Millipore, United States) and of O_3_ with an O_3_ scrubber made by a copper tube connected in the Filter paper (47 mm, Whatman, Germany) coated with KI (10%) to prevent oxidant interferences inside the Teflon bags. An oil-free compressor (Shultz, Brazil) was used to pump the filtered ambient air into Bags. The airflow to each Teflon bag was monitored by an in-line flow meter (flow, 10 L min^−1^). The flow was controlled by a needle valve and adjusted to 2 L min^−1^ for the Teflon bag. The temperature and relative humidity were measured inside Telfon Bags using a commercial sensor (Model Rotronics YA-100F, Walz, Germany). The BVOCs were collected in cartridges containing 100 mg of Tenax TA mesh 60/80, coupled to one of the openings, and associated with a suction pump with airflow of 0.2 L min^−1^ and a total of 1.5 L min^−1^ of inserted air (total of 90 min sampling). The samples were stored in a refrigerator for further chemical analysis.

The sampled BVOCs were analyzed in gas chromatography coupled to mass spectrometry (CG-EM Agilent 5977) and desorbed in nitrogen gas by an automatic thermal desorption system (ATD650 from Perkin-Elmer, Perkin Elmer, Waltham, MA, United States), at 250°C for 5 min, with transfer temperature of 200°C and heating rate of 40°C s^−1^ with cryofocusing injection at −30°C. The total time of the analysis cycle was 80 min. The separation of the gaseous sample was carried out by the HP-5 capillary column (50 m × 0.2 mm i.d. × 0.5 μm film thickness; Hewlett-Packard) using Helium (He) as the carrier gas.

The BVOC identification was performed by comparing the mass spectra of the sample with those contained in the chemical library (Wiley/NIST). Quantification was performed based on the analytical curves of the standards (α-pinene, β-pinene, o-cymene, β-ocimene, α-terpinene, D-limonene, α-copaene, caryophyllene, 3-carene, and humulene) acquired by Sigma Aldrich, commercially available. In addition, the α-Pinene standard curve was adopted to estimate the concentrations of compounds that did not have available standards for their respective curves.

The emission rate [Es (ng·g1·h^−1^)] of each compound was calculated using the following equation adapted from Bracho-Nunez et al. (2013):


$$Es=\Delta c\left(\frac{Q}{dw}\right)$$

where, ∆c (ng L^−1^) is the difference between blank and sample concentration; *Q* (L h^−1^) is the flow, and *dw* (g) is the dry mass of the sample. The data matrix was transformed using [log (x + 1)] to attenuate the variance heterogeneity.

The BVOCs identified were classified into four categories: monoterpenes (MON), sesquiterpenes (SEQ), green leaves compounds (GLV), and other compounds (OTC).

### Data Analyses

A multivariate approach was used to examine the differences in the BVOC profile between the species and understand how O_3_ exposure affects each species. The fourth-root transformed emission rates of each BVOC were used as response variables to build a resemblance matrix based on Bray-Curti’s distance. PERMDISP and PERMANOVA tests were performed with 999 permutations (Anderson, 2017). Both species identity and O_3_ exposure were treated as fixed factors. Pairwise comparisons were used to explore significant factors and interactions further. SIMPER procedure was used to describe the BVOCs of each species and the differences among them, and the effect of O_3_ treatment for each species, which were visually represented using a non-Metric Multidimensional Scaling (nMDS; Clarke, 1993). The SIMPER procedure was used to identify each compound’s contribution and select the most representative ones. Our results focused on the BVOCs that contributed up to 50% of the similarity between individuals or dissimilarity comparing different treatments. All tests were performed with Primer 6.0 software (Clarke and Gorley, 2006).

## Results

### Detection of PCD and H_2_O_2_

**Figure 1:**
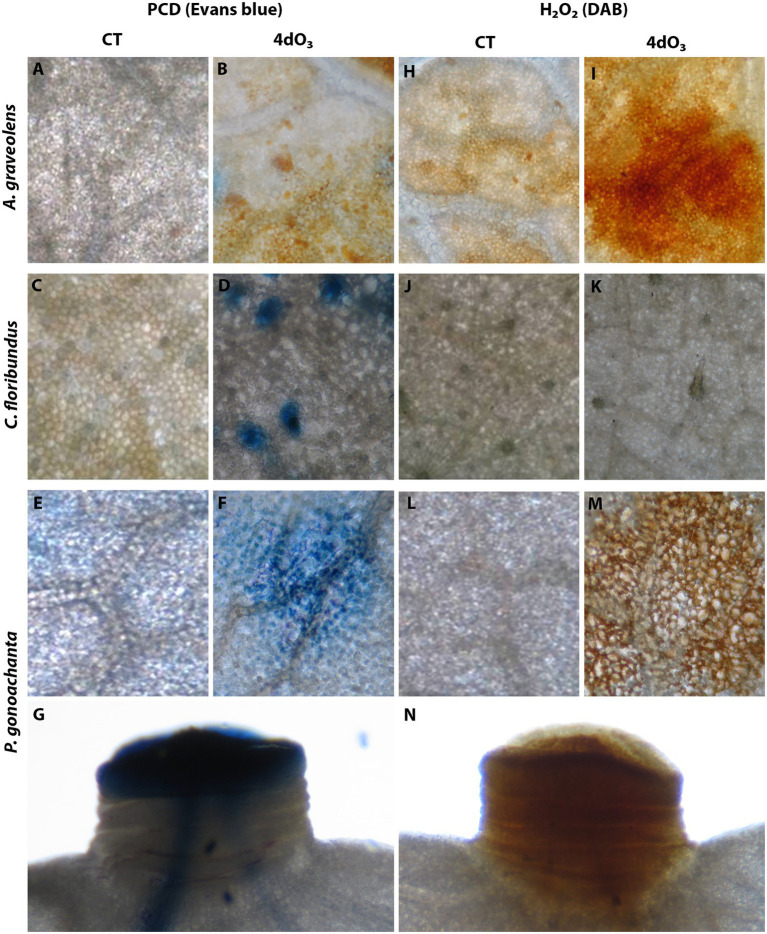
Localization of programmed cell death (PCD) visualized by Evans blue staining. **(A,B)** *Astronium graveolens*, with PCD not observed in CT **(A)** or 4dO_3_ samples **(B)**; **(C,D)** *Croton floribundus,* with PCD, observed in palisade parenchyma cells of 4dO_3_ samples **(D)** but not in CT samples **(C)**; **(E,G)** *Piptadenica gonoacantha* with PCD observed in palisade parenchyma cells of 4dO_3_ samples **(F)** but not in CT samples **(E)** and the leaf pulvinus **(G)**. H_2_O_2_ deposits brown-stained by DAB. **(H,I)** *Astronium graveolens,* with H_2_O_2_ observed in palisade parenchyma cells of 4dO_3_ samples **(I)** but not in CT samples **(H)**; **(J,K)** *C. floribundus,* with H_2_O_2_ not observed in CT **(J)** or 4dO_3_ samples **(K)**; **(L–N)** *Piptadenica gonoacantha* with H_2_O_2_ observed in palisade parenchyma cells of 4dO_3_ samples **(M)** but not in CT samples **(L)** and the leaf pulvinus **(N)**. **A–F** and **H–M**, scale bars = 150 μm. **G,N**, scale bars = 250 μm.

Figure 1 combines the most representative pictures to visualize the accumulation of H_2_O_2_ and PCD for each species analyzed. *Astronium graveolens* did not show PCD in CT or 4dO_3_ samples (Figures 1B vs. 1A), but all 4dO_3_ samples showed H_2_O_2_ accumulation (Figures 1C vs. 1D). PCD results for *C. floribundus* were doubtful since leaves have a heavy trichome layer that hindered stain penetration into the leaf tissues (Figures 1F vs. 1E), and therefore, H_2_O_2_ was not detected in any sample (Figures 1H vs. 1G). In *P. gonoachanta*, PCD occurred in restricted areas of palisade parenchyma and around the stomata guard cells in 4dO_3_ samples but not in CT (Figures 1J vs. 1I). All 4dO_3_ samples accumulated H_2_O_2_ accumulation, while CT samples did not accumulate (Figures 1L vs. 1K). In this species, PCD and H_2_O_2_ were also evident in the leaf pulvinus (Figures 1M,N, respectively).

### Constitutive BVOC Emission Profile

The three species studied emitted a very diverse BVOC composition, classified into four classes: monoterpenes (MON), sesquiterpenes (SEQ), green leaf volatiles (GLV), and other compounds (OTC). The contribution of all components as identified by the SIMPER procedures to show the similarity/dissimilarity across species and treatments is available in S1 and S2.

**Figure 2:**
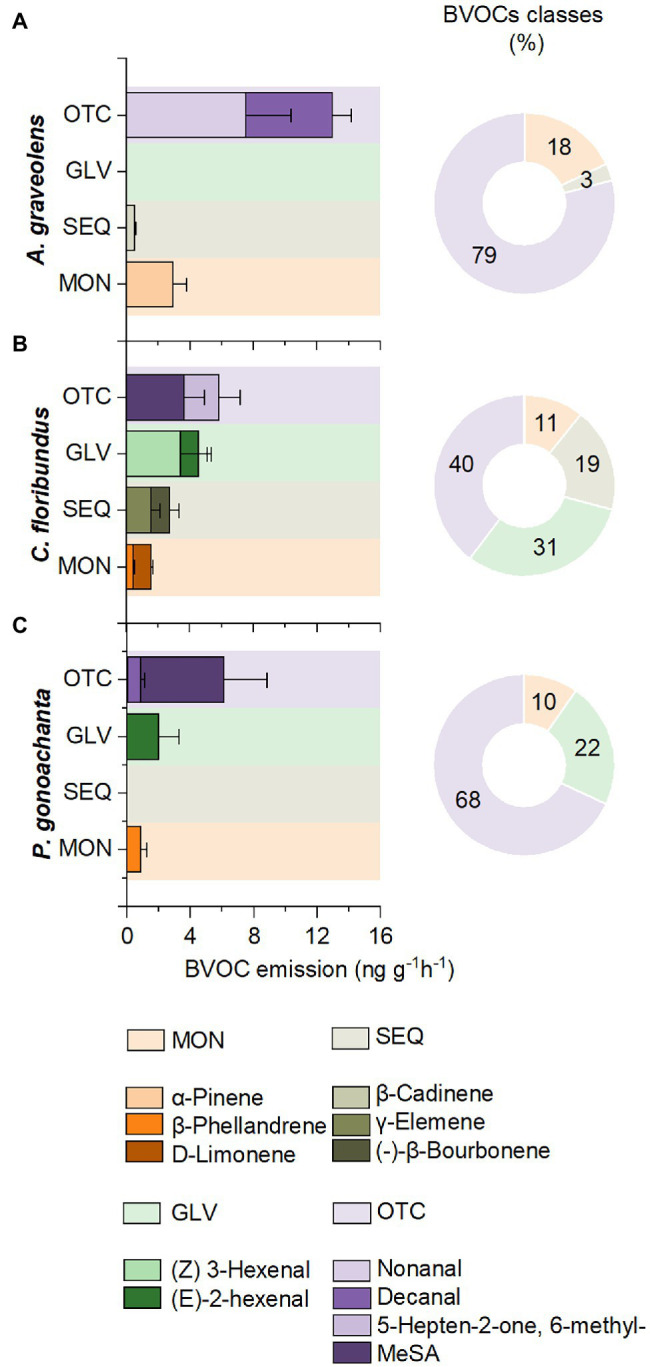
Constitutive emission rates and percentage of different classes of biogenic volatile organic compounds (BVOCs): monoterpenes (MON), sesquiterpenes (SEQ), green leaf volatiles (GLV), and other compounds (OTC) in seedling of **(A)** *Astronium graveolens*, **(B)** *Croton floribundus*, and **(C)** *Piptadenica gonoacantha* exposed to control conditions (CT). Data are mean ± standard error.

Based solely on CT samples, the constitutive compounds emitted by each species were evaluated. The similarity in constitutive compounds between *A. graveolens* individuals was 62%, with four compounds contributing to 50% of the similarity (S1). Considering the emission rate of these compounds, the most abundant constitutive compounds in *A. graveolens* were OTC (nonanal and decanal), which accounted for 79% of the total emission. Therefore, α-pinene (MON) had an actual constitutive emission rate contributing 18% of the total emission, while β-Cadiene was the most representative SEQ but contributed only 3% of the emission (Figure 2A).

Individuals of *C. floribundus* showed a more uniform composition of constitutive compounds than *A. graveolens*, with a 76% similarity between individuals. As shown in S1, eight compounds were responsible for 54% of the similarity. The emission rate of different compound classes was more homogeneous for this species. The OTC class had the highest emission rate contributing to 40% of the most representative compound classes emitted, characterized by 5-Hepten-2-on,. 6-metil-, and Methyl Salicylate (MeSA). The GLV was the second class of compounds with the highest emission rates (31% of contribution) characterized by (Z) 3-hexenal, and (E)-2-hexenal. The SEQ represented 19% of the total emission, represented by γ-elemene and (−)-β-bourbonene. The MONs were the less representative compounds (11% of contribution), characterized by β-phellandrene and D-limonene (Figure 2B).

Individuals of *P. gonoacantha* showed the lowest similarity (61%) with only four most representative compounds responsible for 57% of the similarity (S1). In addition, the emission rate of OTC compounds for this species was the most representative, mainly represented by the MeSA. The GLV compounds were the second class of compounds with the highest emission rate, contributing 22% of the total emission and represented by (E)-2-hexenal. The MONs were the less representative compounds (11% of contribution), characterized by β-phellandrene. This species did not emit SEQ (Figure 2C).

### Effect of O_3_ on the BVOC Emission Profile

**Figure 3:**
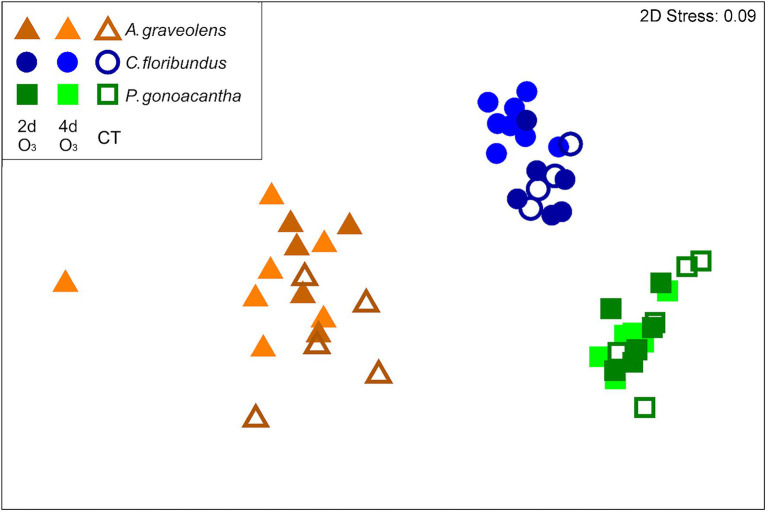
Non-metric multidimensional scaling (nMDS) representation of the SIMPER procedure, used to describe the BVOCs of each species and the effect of O_3_ treatment on each species.

The emission rates of BVOC affected by O_3_ are shown in Table 1. The MON in *A. graveolens* were the most affected species, with a high level of β-myrcene, α-terpinene, and D-limonene, while O_3_ most shifted the level of SEQ and OTC in *C. floribundus*, and GLV and OTC in *P. gonoachanta*. Also, O_3_ induced the production of α-ionone and trans-β-ionone in *C. floribundus*. Moreover, individuals exposed to distinct O_3_ treatments had similar distance to the centroid (PERMDISP F_2,50_ = 2.04; *p* = 0.20). *A. graveolens* individuals showed a more variable BVOC profile than the other two species (PERMDISP F_2,50_ = 9.19; *p* = 0.02, Figure 3). Species differences determined how O_3_ exposure affected the BVOC profile, resulting in a species identity by interaction with the O_3_ treatment (Table 1). In *A. graveolens*, the O_3_ exposure resulted in a completely distinct BVOC profile from CT individuals regardless of the exposure time (2dO_3_ or 4dO_3_). For this species, the oxidative burst cascades, known as the first step in plant response to O_3_ stress, were detected in terms of H_2_O_2_. However, due to the low light intensity inside the experimental facility, PCD, which is the final result of the O_3_ degenerative process, was not observed for this specie once the photo-oxidative and the O_3_ stress have been demonstrated to act synergistically to trigger a hypersensitive-like response (HR-like) processes for this species (Moura et al., 2018).

**Table 1:** Summary results of PERMANOVA and *post-hoc* tests for BVOC profile considering the effects of species identity and O_3_ treatment.

Source	df	MS	F		*p*	
Species id	2	31245.0	48.90		**0.001**	
O_3_ treat	2	1177.5	1.84		**0.018**	
S x O	4	1361.2	2.13		**0.001**	
Error	44	638.9				
*Post-hoc* tests	2dO_3_ vs. 4dO_3_		2dO_3_ vs. CT		4dO_3_ vs. CT_3_	
**Species**	t	*p*	t	*p*	t	*p*
*A. graveolens*	0.92	0.59	1.62	**0.02**	1.63	**0.03**
*C. floribundus*	2.36	**0.02**	1.94	**0.02**	0.53	0.94
*P. gonocantha*	1.07	0.36	1.23	0.22	0.69	0.69

In contrast, when *C. floribundus* was exposed to 2dO_3_, its BVOC profile was distinct from CT individuals; however, a prolonged exposure (4dO_3_) did not change the BVOC profile. The O_3_ exposure did not affect BVOC production by *P. gonocantha*.

**Figure 4:**
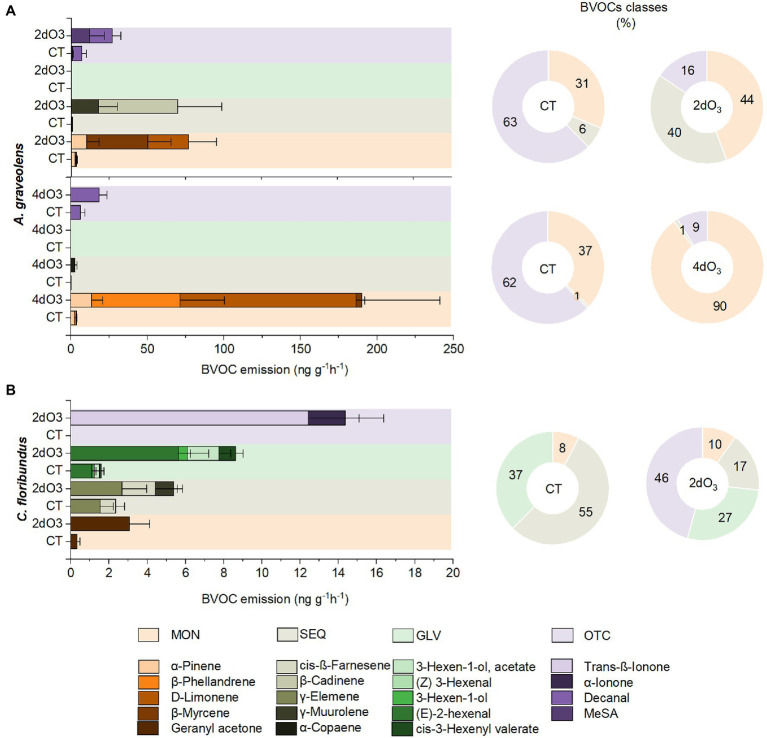
Constitutive emission rates and percentage of different classes of BVOCs: MON, SEQ, GLV, and OTC in seedlings of **(A)** *Astronium graveolens* and **(B)** *Croton floribundus* exposed to CT and filtered air enriched with 80 ppb of ozone for 2 (2dO_3_) and 4 days (4dO_3_). Data are mean ± standard error.

In *A. graveolens*, seven compounds were identified as the most significant in differentiating CT from 2dO_3_ individuals (52% of the contribution, S2). Regarding the emission of these compounds, there was a remarkable increase in the MON proportion associated with a higher emission rate of α-pinene and β-myrcene and the emergence of D-limonene. The SEQ proportion also increased and was related to γ-muurolene and β-cadinene emission. However, there was a proportional decrease in OTC emission compared to the OTC. The emission rate of decanal (OTC) and MeSA was higher in 2dO_3_ than in CT (Figure 4A; Supplementary Figure S2). Exposure to 4dO_3_ resulted in a higher concentration of the six BVOCs that most contributed to differentiating the individuals in this group from the CT group (S2). In this case, the MON emission rate was even more noticeable, contributing to 90% of the total emission, and was associated with a higher emission of α-pinene, β-phellandrene, β-myrcene, and D-limonene. The SEQ proportion did not vary between treatments, but the emission of α-Copaene substantially increased in 4dO_3_ plants. Although the proportional OTC emission was reduced, the decanal (OTC) emission rate was higher in 4dO_3_ than in CT (Figure 4A; Supplementary Figure S2).

In *C. floribundus*, 10 BVOCs mainly contributed to discriminating individuals exposed to 2dO3 from those in the CT group (S2). The MON proportion increased only 2% compared to the OTC; however, the emission of Geranyl acetone was much higher in 2dO_3_ than in CT plants. The SEQ proportion decreased from 55% to 17%; however, the emission rate of γ-elemene, cis-ß-farnesene was still higher in 2dO_3_ than in CT plants, and there was an emergence of α-muurolene. The GLV showed the same pattern with 10% decrease in the proportion of compounds in 2dO_3_ compared to CT plants. All compounds that represented the class [(E)-2-hexenal, 3-hexen-1-ol, 3-hexen-1-ol acetate, cis-3-hexenyl valerate] had a higher emission rate in 2dO_3_ than in CT plants. There was a remarkable increase from 0% to 46% in the proportion of OTC compounds, significantly related to the emergence of the compounds α-Ionone and trans-ß-Ionone by 2dO_3_ individuals (Figure 4B; Supplementary Figure S2).

## Discussion

### Variability and Differences in BVOC Profiles

The synthesis of chemical products by plants represents an investment in energy and resources for the organisms and is directly influenced by the species resource use strategy.

The BVOC, in particular MON and SEQ, are usually sequestered in complex, multicellular secretory structures, and so storage costs for these substances are also likely to be substantial. However, not all types of BVOCs require large investments of resources for accumulation. For instance, the maintenance of SEQ pools is probably less expensive than MON and GLV because there is no evidence that substantial quantifies of SEQ are lost due to metabolic turnover, volatilization, or leaching.

Plants may reduce their net BVOC costs by employing individual compounds in more than one role or by catabolizing substances that are no longer needed. In the light of investment in the complexity of BVOC production and costs is likely explained that plant volatile is a potentially functional trait (i.e., a character that affects fitness and survival) with variability and high dependence on the plant species and its defenses as well as the type of stress received (Holopainen and Gershenzon, 2010).

The concept of plant defense involves a range of traits and defense compounds, particularly the constitutive BVOC is considered as a plant ecology strategy (Onoda et al., 2017). Thus, according to the plant economic spectrum theory, the acquisitive species, recognized for their rapid growth, high nutrition quality, and an increased level of carbon fixation, provide resource factors for the likely increase of BVOCs compared to conservative ones.

Given the species resource use strategy, *C. floribundus* and *A. graveolens* can be considered acquisitive species (Campos, 2020), while *P. gonoachanta* exhibits a conservative strategy (greater height, longer lifespan, low specific leaf area, low nitrogen and phosphorus levels in leaves, low photosynthetic rate, and high wood density) with slower growth and resource use (Esposito et al., 2018; Campos, 2020). Subsequently, we would expect that acquisitive species, which assimilate carbon quickly for rapid growth, are likely to store organic compounds structurally more complex with higher carbon numbers as SEQ (Brandes et al., 2016).

Previous studies indicated *C. floribundus* as an SEQ-emitter, being γ-elemene and—(−) β-bourburne its markers (Cardoso-Gustavson et al., 2014; Pedrosa et al., 2020). However, in the present study, the OTC class of compounds was more representative, but the SEQ γ-elemene and—(−) β-bourburne were the most important to characterize the specie. These findings are according to the fast acquisitive strategy of resources adopted by plants in conditions of large resources available and lesser environmental stress (Pellegrini, 2012; Wigley et al. 2016).

*A. graveolens* was less acquisitive than *C. floribundus*; therefore, we expected a lower SEQ emission, as confirmed by our results. Moreover, *A graveolens* can be considered as a MON-emitter, especially of α-Pinene, but also with high rates of OTC compounds, such as Decanal and Nonanal.

It is well-known that *P. gonoachanta* has long-lived leaves and a high cost and slow return on investment in carbon and nutrients (Campos, 2020; Teixeira et al., 2020). Therefore, this species often shows high leaf dry matter content, low leaf thickness, low photosynthetic rates, and low nitrate reductase activity (Reich et al., 2014). Generally, this species considered conservative has low metabolic activity, investing in the formation of stiffer and more rigid and robust structures, such as leaves and dense, lignified stems (Reich et al., 2014; Domingos et al., 2015). Herbivory is highly damaging to conservative species, which usually invest in BVOCs to defend against biotic and abiotic stresses. The emission of BVOC in leaves damaged by herbivory was 2.5 times greater than in intact leaves, representing a plastic phenotypic response and acts as a primer in undamaged plants (Maffei, 2010; Wölwer-Rieck et al., 2014; Hu et al., 2021).

The constitutive GLV emitted by *P. gonoachanta* (CT exposure) could represent an investment in induced defenses, indicating the onset of an “alert” state and an accelerated response in case of attack by herbivores (Engelberth et al., 2004; Maffei, 2010). Therefore, most GLV might indicate the occurrence of chemical communication processes, preparing the healthy plant for a future herbivorous attack (Hu et al., 2021). The accumulation of H_2_O_2_ and PCD detected in the pulvinus of *P. gonoachanta* is directly related to intense defoliation (Moura et al., 2014, 2018), which may be a consequence of the GLV emission, produced by H_2_O_2_ signaling and lipoxygenases mechanisms (LOX) that induce plant defenses (Tian et al., 2019). Furthermore, under stress conditions, the H_2_O_2_ accumulation has been demonstrated to be closely related to foliar abscission (Sakamoto et al., 2008), acting as a signaling molecule able to elicit PCD (Gechev and Hille, 2005).

### O_3_ Effect on BVOC Responses

The BVOC responses to O_3_ depend on the plant species, its oxidative stress tolerance capacity (Vickers et al., 2009; Loreto et al., 2014), and the O_3_ uptake. The latter is a consequence of stomatal opening and optimal photosynthesis conditions. The stomata flux is considered the most reliable index of potential O_3_ damage (Yuan et al., 2017), and it has been reported to be strongly related to PCD and H_2_O_2_ accumulations (Moura et al., 2014, 2018). In the present study, the PCD and H_2_O_2_ were less intense in *C. floribundus* and *A. graveolens* suggesting that the higher O_3_ uptake and subsequent oxidative stress increased in *P. gonoachanta*. However, the O_3_ affected all species’ total BVOC emission rate, particularly the induction of *de novo* and constitutive chemical compounds in *A. graveolens* and *C. floribundus*. Our results confirmed that high O_3_ increases the total BVOC emissions and induces specific compounds for each species in different ways after 2dO_3_ and 4dO_3_ exposure, in particular increase of MON in *A. graveolens* and SEQ in *C. floribundus*.

The SEQ, for example, is exceptionally reactive with O_3_ and its reactivity reflects the ability of the plants to prevent oxidative damage by quenching harmful reactive oxygen species within plants or their headspace. Furthermore, SEQ might mitigate damage even more effectively than isoprene and MON. For example, the (E)-β-caryophyllene, is 43 times more reactive with O_3_ than is the D-limonene (Shu and Atkiinson, 1994). According to Loreto and Schnitzler (2010), the emission patterns hint that these compounds might ameliorate oxidative stress: sesquiterpene emission more than isoprene and MON emission can increase in response to oxidative stress (Loreto et al., 2014) by increasing the vapor pressure of already-present compounds *via* increased temperature or altering stomatal conductance. In addition, while MON shown to enhance abiotic stress tolerance is synthesized in plastids, SEQ are generally synthesized in the cytosol, which could constrain their roles in protection against localized oxidative stress in plastids. In *A. graveolens*, the increase of MON compounds, especially the emergence of D-Limonene (MON) and the increased emission of β-Cadiene (SEQ), Decanal (OTC), and MeSA (OTC), can be considered markers of the 2dO_3_ effect, whereas the cumulative damage for 4dO_3_ was marked by the increase of MON compounds, including α-Phellandrene (MON), and a substantial increment in the D-Limonene (MON) emission rate. Unlike *A. graveolens*, BVOC emission by *C. floribundus* was affected only on 2dO_3_ exposure, particularly marked by the emergence of *de novo* compounds, such as α-muurolene (SEQ), trans-β-ionone (OTC), α-ionone (OTC), and by the emission increase of geranyl acetone (MON), cis-β-farnesene (SEQ), γ-elemene (SEQ), and several GLV compounds, what could be explained by the higher tolerance of *C. floribundus* to ozone stress, which is able to produce the novo compounds with the chemical potential to sequester reactive oxidative species (ROS) from cell, reduzing the oxidative stress.

Most individuals that enhanced SEQ were found in *C. floribundus*. In particular, the emergence of α-muurolene could function as a trigger against oxidative stress in this species, suggesting that it is an indicator of the damage onset. However, the emission rate of cis-β-farnesene (SEQ) and γ-elemene (SEQ) increased with O_3_ exposure.

Furthermore, in exposed individuals of *C. floribundus*, there was also the emergence of α-Ionone (OTC) and trans-ß-ionone (OTC). It is interesting to note that trans-β-ionone has been produced in arabidopsis leaves exposed to oxidative stress, and it was able to induce some H_2_O_2_ accumulation (Ramel et al., 2012), which is considered as a marker of stress tolerance. SEQs and OTC present higher antioxidant potential than MON once their chemical structures are more reactive to O_3_ and have hormone-like properties. Among them, cis-β-farnesene (SEQ), α-ionone (OTC), and MeSA (OTC) act as phytohormones and are functional in plant communications (Bunsick et al., 2021). The compounds cis-β-farnesene and α-ionone have a high potential to protect plants against abiotic stress; they have a rate constant for reactivity with O_3_ on the same order of magnitude as caryophyllene, and they might mediate O_3_ stress tolerance (Palmer-Young et al., 2015). In addition, MeSA is one of the critical messenger molecules synthesized by plants in response to stress. This compound may act as a mobile signal throughout the plant. It triggers the systematic acquired resistance through its precursor, the salicylic acid, enhancing chemical defenses, such as antioxidants (Cardoso-Gustavson et al., 2014). Interestingly, in the present study, MeSa was not significantly affected in *C. floribundus*. However, in one of our previous experiments (Cardoso-Gustavson et al., 2014), MeSa increased when *C. floribundus* individuals were subjected to 7dO_3_ exposure, demonstrating that in this species, the O_3_ effect might be time exposure-dependent.

The MeSA variations followed the inverse pattern of cis β-Farnesene and β-Ionone for *C. floribundus*, while in *A. graveolens*, its levels increased after 2dO_3_. Based on these results we hypothesized that BVOCs would act as signaling compounds in primary defense mechanisms with greater intensity in *C. floribundus* than in *A. graveolens*, preparing the species for future stress (Hu, 2022). PCD and H_2_O_2_ results may support this hypothesis since O_3_ could promote H_2_O_2_ accumulation in *A. graveolens*. However, it did not exceed the toxic levels to induce PCD, once this event was rarely detected on O_3_ experimental samples. Noteworthy, O_3_ could not induce H_2_O_2_ and PCD in *C. floribundus*. Its tolerance has also been related to various factors, including the numerous trichomes on the abaxial surface that protect its stomata and act as a barrier against the uptake of gaseous pollutants (Evert, 2006; Dias et al., 2019), its powerful antioxidative capacity against ROS formation (Domingos et al., 2015), and most likely, to paramount signaling volatiles (Cardoso-Gustavson et al., 2014).

Interestingly, *P. gonoachanta* induced many compounds that act in chemical defenses, such as the MeSA (OTC) (E)-2-hexenal. However, the O_3_ stress did not affect the BVOC emission in this species. In *P. gonoachanta*, H_2_O_2_ accumulation was strongly detected and directly related to a HR-like response. This type of response is well described as an O_3_ effect and consists in the collapse of palisade parenchyma cells (Vollenweider et al., 2003; Moura et al., 2014). The fast structural response of this species may be another reason for its O_3_ sensibility reported in the previous studies.

The emission of O_3_-induced BVOC into the atmosphere can provide changes in ecological and atmospheric perspectives (Pinto et al., 2010) From an ecological point of view, the changes in the BVOC profile affect their multiple functions that protect plants from biotic and abiotic stressors, inhibiting germination and growth of neighboring plants and thus decreasing competition, disturbing the tritophic and plant–plant interaction. Whereas both constitutive or induced BVOC interfere in the biosphere-atmosphere interactions as potential precursors of atmospheric oxidants and even secondary aerosols (Abis et al., 2021), impacting the regional radiative forcing.

Based on our results and previous research on the BVOCs emission from the species studied (Cardoso-Gustavson et al., 2014; Pedrosa et al., 2020), we consider that *A. graveolens*, *C. floribundus*, and *P. gonoacantha* are significant emitters of MON, SEQ, and OTC, respectively. The effect of O_3_ on BVOC response can shift the emission profile, increasing the levels of compounds that mediate the abiotic stress tolerance, particularly in *C. floribundus*, where the SEQ levels were most affected. The BVOC emission can also affect the atmospheric chemistry; MON has more impact on O_3_ and particle formation than SEQ and OTC, suggesting that the potential use of *C. floribundus* in urban reforestation is not compromising the air pollution.

## Conclusion

This study is the first to analyze the BVOC emission from leaf blades and the response to O_3_ stress in individuals of *A. graveolens*, *C. floribundus*, and *P. gonoacantha*, three species native to the Atlantic Forest. The BVOC emission in combination with histochemical techniques may suggest that *C. floribundus* as the most O_3_ tolerant species, followed by *A. graveolens* and then *P. gonoachanta*, which was the most sensitive, showing no response in BVOC emission. Thus, our results suggest that the quality and quantity of BVOC emission seem to be associated with strategies of species in protecting against oxidative stress.

Furthermore, the three species are widely used for afforestation of urban areas and reforestation of degraded areas. They release BVOCs that interact with the atmospheric chemistry and generate environmentally important by-products, particularly the tropospheric O_3_ formation, which can also induce BVOCs.

## Data Availability Statement

The original contributions presented in the study are included in the article/Supplementary Material, and further inquiries can be directed to the corresponding authors.

## Author Contributions

VB and MP performed the measurements. BM designed and planned histochemical analysis. GD designed and performed the statistical analysis. SS designed, planned, and supervised the work. BM, GD, and SS wrote the manuscript. All authors contributed to the article and approved the submitted version.

## Funding

The authors would like to thank the Fundação de Amparo à Pesquisa do Estado de São Paulo (FAPESP 2016/25109-3 and 2012/11662-8) and the Conselho Nacional de Desenvolvimento Cientifico e Tecnologico (CNPq, 3055395/2019-0).

## Conflict of Interest

The authors declare that the research was conducted in the absence of any commercial or financial relationships that could be construed as a potential conflict of interest.

## Publisher’s Note

All claims expressed in this article are solely those of the authors and do not necessarily represent those of their affiliated organizations, or those of the publisher, the editors and the reviewers. Any product that may be evaluated in this article, or claim that may be made by its manufacturer, is not guaranteed or endorsed by the publisher.
