# Supplementary material for: Ozone Impact on Emission of Biogenic Volatile Organic Compounds in Three Tropical Tree Species From the Atlantic Forest Remnants in Southeast Brazil
Source: Front Plant Sci. 2022 Jun 24;13:879039. doi: 10.3389/fpls.2022.879039 (PMC9263830; doi:10.3389/fpls.2022.879039)
Supplement: Supplementary file 1 [file Table_5.DOCX]

S1. Summary results of SIMPER analyses showing which Biogenic Volatiles Organic Compounds (BVOC) contribute to up to 90% of the similarity between the individuals of a) *Astronium graveolens*; b) *Croton floribundus* and C) *Piptadenia gonoachanta*. Values are the fourth root transformed abundance of species, contribution (Contrib. %) and cumulative contribution (Cum. %).

| a) *A. graveolens* - Average similarity:62.06 | | | |  |  |
| --- | --- | --- | --- | --- | --- |
| **Species** | **av. abundance** | **av. similarity** | **Sim/SD** | **Contribution%** | **Cummulative%** |
| Nonanal | 1.5 | 11.74 | 6.14 | 18.92 | 19 |
| Decanal | 1.36 | 7.42 | 1.14 | 11.96 | 31 |
| ß-Cadinene | 0.81 | 6.18 | 6.56 | 9.96 | 41 |
| α-Pinene | 1.09 | 6.14 | 1.14 | 9.9 | 51 |
| Caryophyllene | 0.7 | 4.94 | 6.41 | 7.96 | 59 |
| Geranyl acetone | 0.93 | 4.63 | 1.14 | 7.46 | 66 |
| 5-Hepten-2-one6-methyl- | 0.7 | 4.22 | 1.08 | 6.79 | 73 |
| α-Phellandrene | 0.56 | 3.11 | 1.07 | 5.01 | 78 |
| o-Cymene | 0.74 | 2.8 | 1.12 | 4.51 | 82 |
| ß-Myrcene | 0.72 | 2.37 | 0.62 | 3.82 | 86 |
| γ-Muurolene | 0.52 | 2.07 | 0.6 | 3.34 | 90 |
| Humulene | 0.39 | 2.03 | 1.14 | 3.27 | 93 |
|  | | | |  |  |
| b) *C. floribundus* - Averagesimilarity:76.15 | | | |  |  |
| **Species** | **av. abundance** | **av. similarity** | **Sim/SD** | **Contribution%** | **Cummulative%** |
| Decanal | 1.31 | 6.76 | 5.49 | 8.88 | 9 |
| D-Limonene | 1.02 | 5.78 | 12.54 | 7.6 | 16 |
| (Z)3-Hexenal | 1.2 | 5.63 | 7.2 | 7.39 | 24 |
| γ-Elemene | 1.06 | 5.38 | 8.8 | 7.07 | 31 |
| Methylsalicylate | 1.06 | 4.92 | 10.35 | 6.45 | 37 |
| (-)-ß-Bourbonene | 0.94 | 4.51 | 14.7 | 5.93 | 43 |
| ß-Phellandrene | 0.79 | 4.27 | 15.49 | 5.61 | 49 |
| (E)-2-hexenal | 0.91 | 4.2 | 3.67 | 5.52 | 54 |
| ß-Copaene | 0.75 | 3.8 | 8.38 | 4.99 | 59 |
| Nonanal | 0.64 | 3.53 | 11.75 | 4.63 | 64 |
| 5-Hepten-2-one6-methyl- | 0.6 | 3.25 | 8.94 | 4.26 | 68 |
| (-)-Spathulenol | 0.64 | 3.14 | 11.33 | 4.13 | 72 |
| Caryophyllene | 0.59 | 3.05 | 5.67 | 4 | 76 |
| ß-Ocimene | 0.63 | 2.88 | 7.65 | 3.78 | 80 |
| α-Copaene | 0.54 | 2.86 | 12.38 | 3.76 | 84 |
| Humulene | 0.43 | 2.33 | 9.7 | 3.07 | 87 |
| Geranyl acetone | 0.59 | 2.19 | 0.91 | 2.88 | 90 |
| cis-ß-Farnesene | 0.71 | 2.1 | 0.88 | 2.76 | 93 |
|  |  |  |  |  |  |
| *P. gonoacantha* - Averagesimilarity:61.65 | | | |  |  |
| **Species** | **av. abundance** | **av. similarity** | **Sim/SD** | **Contribution%** | **Cummulative%** |
| Decanal | 0.93 | 9.92 | 3.23 | 16.09 | 16.09 |
| (E)-2-hexenal | 0.94 | 7.34 | 5.21 | 11.9 | 28 |
| ß-Phellandrene | 0.83 | 7.08 | 3.68 | 11.49 | 39.48 |
| Methylsalicylate | 1.08 | 5.65 | 1.08 | 9.17 | 48.65 |
| 5-Hepten-2-one6-methyl- | 0.46 | 5.02 | 2.88 | 8.14 | 56.8 |
| Linalool | 1.06 | 4.97 | 1 | 8.06 | 64.86 |
| Nonanal | 0.45 | 4.91 | 3.43 | 7.96 | 72.82 |
| ß-Ocimene | 0.52 | 3.85 | 2.87 | 6.25 | 79.07 |
| Octanal | 0.41 | 3.39 | 0.98 | 5.49 | 84.56 |
| α-Farnesene | 0.99 | 3.02 | 0.54 | 4.89 | 89.45 |
| (Z)3-Hexenal | 0.83 | 2.82 | 0.6 | 4.58 | 94.04 |
