# Supplementary material for: Ozone Impact on Emission of Biogenic Volatile Organic Compounds in Three Tropical Tree Species From the Atlantic Forest Remnants in Southeast Brazil
Source: Front Plant Sci. 2022 Jun 24;13:879039. doi: 10.3389/fpls.2022.879039 (PMC9263830; doi:10.3389/fpls.2022.879039)
Supplement: Supplementary file 2 [file Table_6.DOCX]

S2. Summary results of SIMPER analyses showing which volatiles contribute to the dissimilarity between significant levels of O_3_ treatment up to 90%, fourth root transformed abundance of species, contribution (%) and cumulative contribution (%), for a) *Croton floribundus* and b) *Astronium graveolens*

a) *C. floribundus -* Between 2dO_3_ and CT - Average dissimilarity = 33.60

| **Species** | **2dO_3_** | **CT** | **Contribution (%)** | **Cummulative (%)** |
| --- | --- | --- | --- | --- |
|  | **av abundance** | **av abundance** |  |  |
| Trans-ß-Ionone | 1.61 | 0.00 | 11.27 | 11.27 |
| α-Ionone | 0.91 | 0.00 | 6.43 | 17.71 |
| Geranyl acetone | 1.12 | 0.59 | 4.83 | 22.53 |
| 3-Hexen-1-ol, acetate | 0.87 | 0.42 | 4.79 | 27.33 |
| γ-Elemene | 0.83 | 1.06 | 4.75 | 32.08 |
| cis-ß-Farnesene | 0.62 | 0.71 | 4.67 | 36.74 |
| α-Muurolene | 0.63 | 0.00 | 4.40 | 41.14 |
| (E)-2-Hexenal | 1.44 | 0.91 | 4.11 | 45.26 |
| 3-Hexen-1-ol | 0.65 | 0.20 | 4.02 | 49.27 |
| cis-3-Hexenyl valerate | 0.72 | 0.33 | 4.01 | 53.28 |
| Octanal | 0.47 | 0.40 | 3.83 | 57.11 |
| Decanal | 1.46 | 1.31 | 3.81 | 60.92 |
| 1-Octen-3 ol | 0.61 | 0.32 | 3.25 | 64.18 |
| Methyl salicylate | 1.29 | 1.06 | 3.15 | 67.33 |
| 5-Hepten-2-one, 6-methyl- | 1.03 | 0.60 | 3.01 | 70.33 |
| (Z) 3-Hexenal | 1.41 | 1.20 | 2.95 | 73.28 |
| ß-Phellandrene | 0.79 | 0.79 | 2.93 | 76.22 |
| (-)-ß-Bourbonene | 0.97 | 0.94 | 2.91 | 79.12 |
| D-Limonene | 1.29 | 1.02 | 2.69 | 81.81 |
| (-)-Spathulenol | 0.80 | 0.64 | 2.60 | 84.40 |
| n-Hexyl salicylate | 0.41 | 0.25 | 2.53 | 86.94 |
| ß-Copaene | 0.65 | 0.75 | 2.39 | 89.32 |
| Geranyl benzoate | 0.27 | 0.38 | 2.18 | 91.50 |

b) *A. graveolens -* Between 2dO_3_ and CT - Average dissimilarity = 49.58

| **Species** | **2dO_3_** | **CT** | **Contribution (%)** | **Cummulative (%)** |
| --- | --- | --- | --- | --- |
|  | **av abundance** | **av abundance** |  |  |
| ß-Myrcene | 2.23 | 0.72 | 10.74 | 10.74 |
| ß-Cadinene | 1.72 | 0.81 | 9.14 | 19.88 |
| γ-Muurolene | 1.59 | 0.52 | 7.99 | 27.87 |
| Decanal | 1.47 | 1.36 | 6.56 | 34.43 |
| α-Pinene | 1.05 | 1.09 | 6.37 | 40.80 |
| Methyl salicylate | 1.06 | 0.56 | 6.09 | 46.89 |
| D-Limonene | 0.88 | 0.00 | 5.69 | 52.58 |
| α-Phellandrene | 0.75 | 0.56 | 4.46 | 57.04 |
| 5-Hepten-2-one, 6-methyl- | 0.73 | 0.70 | 4.33 | 61.37 |
| Geranyl acetone | 1.14 | 0.93 | 4.13 | 65.51 |
| Citral | 0.70 | 0.29 | 4.05 | 69.56 |
| Nonanal | 1.60 | 1.50 | 4.04 | 73.61 |
| o-Cymene | 0.67 | 0.74 | 3.92 | 77.52 |
| Caryophyllene | 0.59 | 0.70 | 3.57 | 81.10 |
| α-Copaene | 0.59 | 0.27 | 3.48 | 84.58 |
| α-Terpinene | 0.52 | 0.00 | 3.32 | 87.90 |
| Humulene | 0.70 | 0.39 | 3.09 | 90.99 |

Between 4dO_3_ and CT - Average dissimilarity = 49.28

| **Species** | **4dO_3_** | **CT** | **Contribution (%)** | **Cummulative (%)** |
| --- | --- | --- | --- | --- |
|  | **av abundance** | **av abundance** |  |  |
| D-Limonene | 2.19 | 0.00 | 14.59 | 14.59 |
| ß-Myrcene | 2.52 | 0.72 | 13.80 | 28.40 |
| α-Pinene | 1.27 | 1.09 | 7.97 | 36.37 |
| α-Phellandrene | 0.91 | 0.56 | 6.02 | 42.39 |
| Decanal | 2.01 | 1.36 | 5.72 | 48.11 |
| α-Copaene | 0.74 | 0.27 | 5.27 | 53.39 |
| Methyl salicylate | 0.75 | 0.56 | 5.10 | 58.49 |
| Geranyl acetone | 1.53 | 0.93 | 5.02 | 63.52 |
| γ-Muurolene | 0.56 | 0.52 | 4.80 | 68.31 |
| o-Cymene | 0.31 | 0.74 | 4.47 | 72.78 |
| β-Pinene | 0.55 | 0.43 | 4.12 | 76.90 |
| Caryophyllene | 1.13 | 0.70 | 3.53 | 80.43 |
| Citral | 0.46 | 0.29 | 3.19 | 83.62 |
| Humulene | 0.65 | 0.39 | 3.16 | 86.78 |
| 5-Hepten-2-one, 6-methyl- | 1.07 | 0.70 | 3.04 | 89.82 |
| (E)-2-Hexenal | 0.38 | 0.16 | 2.94 | 92.76 |
